# Supplementary material for: Acute Poisoning Readmissions to an Emergency Department of a Tertiary Hospital: Evaluation through an Active Toxicovigilance Program
Source: J Clin Med. 2022 Aug 2;11(15):4508. doi: 10.3390/jcm11154508 (PMC9369450; doi:10.3390/jcm11154508)
Supplement: Supplementary file 1 [file jcm-11-04508-s001.zip › jcm-1744739-supplementary.pdf]

Journal of
Clinical Medicine

MDPI

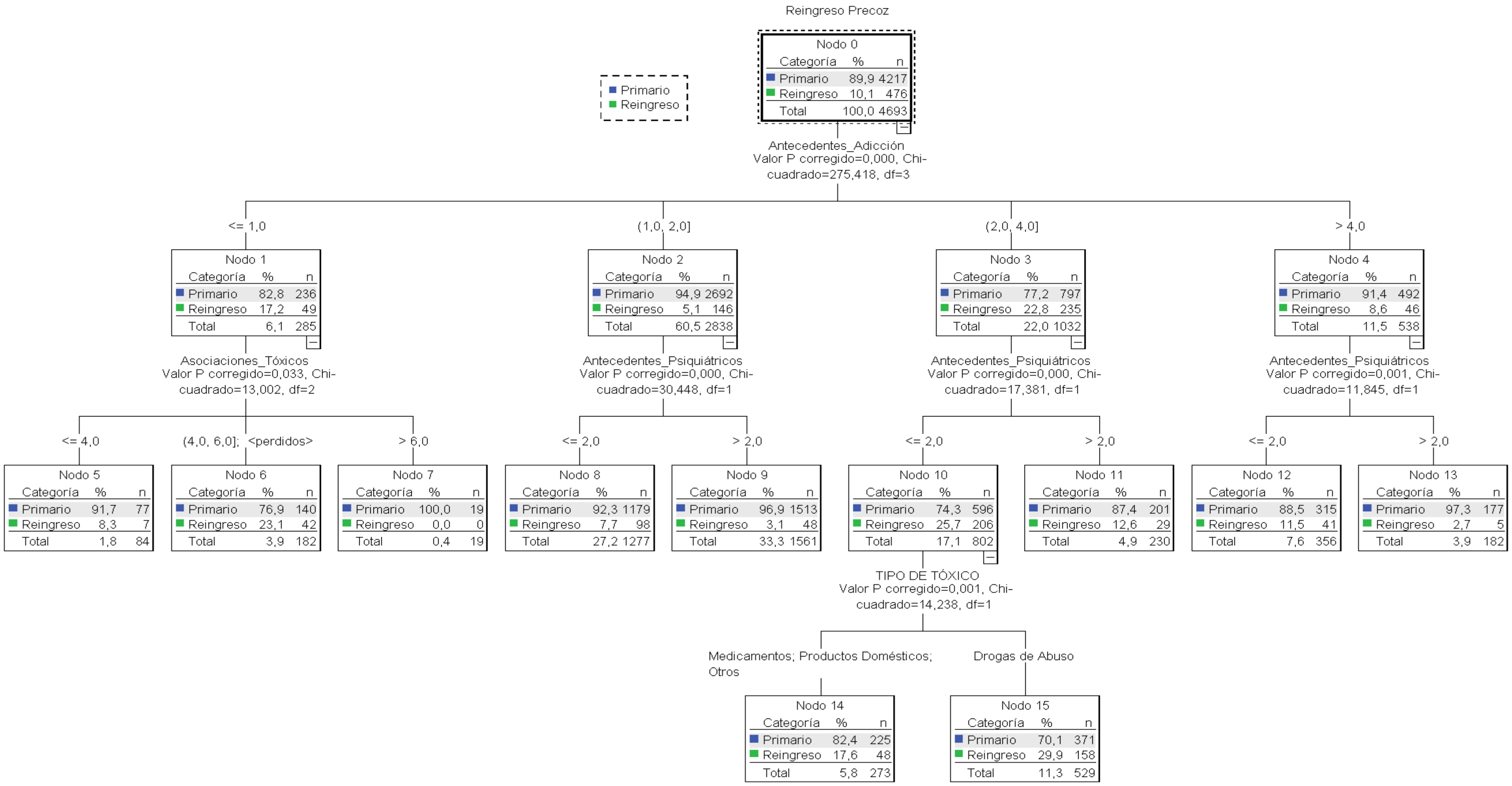

Figure S1. Classification Tree.

Figure Glossary

Valor P corregido: Corrected P-value
Chi-Cuadrado: Chi squared
Tipo de Tóxico: Type of intoxicant
Drogas de Abuso: Drug of abuse
Medicamentos: Medication
Productos Domésticos: Domestic products
Otros: Others
Categoría: Class
Primario: Primary
Reingreso: Readmission
Total: Total
Nodo: Node
Antecedentes Adicción: History of Addiction
Antecedentes Psiquiátricos: Psychiatric History

Table S1. Target category: Readmission.

Earnings for nodes						
Node	Node		Gain		Response	Rating
	N	Percent	N	Percent		
15	529	11,3%	158	33,2%	29,9%	294,5%
6	182	3,9%	42	8,8%	23,1%	227,5%
14	273	5,8%	48	10,1%	17,6%	173,3%
11	230	4,9%	29	6,1%	12,6%	124,3%
12	356	7,6%	41	8,6%	11,5%	113,5%
5	84	1,8%	7	1,5%	8,3%	82,2%
8	1277	27,2%	98	20,6%	7,7%	75,7%
9	1561	33,3%	48	10,1%	3,1%	30,3%
13	182	3,9%	5	1,1%	2,7%	27,1%
7	19	0,4%	0	0,0%	0,0%	0,0%

Growth methods: CHAID

Dependent variable Early Readmission

Risk	
Estimate	Típ. Error
,101	,004

Growth methods: CHAID
Dependent variable: Early Readmission

Clasification			
Observed	Predicted		
	Primary	Readmission	Percent correct
Primary	4217	0	100,0%
Readmission	476	0	0,0%
Overall percent	100,0%	0,0%	89,9%
